# Development of Genetically Modified ARH-77 Feeder Cells for Efficient Expansion of Natural Killer Cells with Potent Anti-Tumor Activity

**DOI:** 10.3390/cancers18111833

**Published:** 2026-06-03

**Authors:** Yu-Jin Lim, Bryan Marr, Safa Ghaziasgar, Cheol-Jung Kim, Yeon-Ju Baek, Geun-Seop Kim, Je-Jung Lee, Yu-Jin Park, Yurim An, Seung-Hwan Lee, Sang-Ki Kim

**Affiliations:** 1 Department of Companion and Laboratory Animal Science, College of Industrial Science, Kongju National University, Yesan 32439, Chungcheongnam-do, Republic of Korea; 2 Department of Biochemistry, Microbiology, and Immunology, Faculty of Medicine, University of Ottawa, Ottawa, ON K1H 8M5, Canada; 3 Department of Applied Biotechnology, Kongju National University, Yesan 32439, Chungcheongnam-do, Republic of Korea; 4 Research Institute for Natural Products, Kongju National University, Yesan 32439, Chungcheongnam-do, Republic of Korea; 5 VaxCell Bio Therapeutics Co., Ltd., Hwasun 58128, Jeollanam-do, Republic of Korea; 6 Department of Hematology-Oncology, Chonnam National University Hwasun Hospital, Hwasun 58128, Jeollanam-do, Republic of Korea; 7 Ottawa Institute of Systems Biology, Centre for Infection, Immunity, and Inflammation, Faculty of Medicine, University of Ottawa, Ottawa, ON K1H 8M5, Canada

**Keywords:** natural killer cells, expansion, ARH-77, feeder cells, immunotherapy

## Abstract

**Background/Objectives:** Adoptive transfer of allogeneic natural killer (NK) cells represents a promising off-the-shelf immunotherapy for cancer, offering advantages in safety and availability over autologous T cell therapies. However, generating therapeutically sufficient NK cell numbers remains challenging due to their low frequency in blood sources. Engineered feeder cell co-cultures have enabled substantial expansions of NK cells to clinically relevant doses. **Methods:** We evaluated the plasma cell leukemia-derived ARH-77 cell line as a feeder for ex vivo NK cell expansion from healthy donor peripheral blood mononuclear cells (PBMCs). Unmodified ARH-77 was compared to K562, followed by engineering both lines to co-express B7-H6 (NKp30 ligand), CD137L (4-1BBL), IL-15, and IL-15Rα via sequential lentiviral transduction. PBMCs were co-cultured with irradiated feeders in cytokine-supplemented (IL-2, IL-21, and later IL-15) RPMI-1640 or DMEM/F-12 medium for up to 28 days. Expansion (fold change in CD3^−^CD56^+^ cells), purity, surface receptor expression, and cytotoxicity (against K562 targets) were quantified. **Results:** Unmodified ARH-77 supported significantly greater NK cell expansion than K562 (model-estimated 681-fold vs. 155-fold at week 4 in RPMI; *p* = 0.0018), with higher purity but comparable cytotoxicity and receptor profiles. Engineered ARH-77 cells achieved robust expansion in RPMI, comparable to that of engineered K562 cells. In optimized DMEM/F-12 medium, engineered ARH-77 drove superior expansion (up to model-estimated 101,241-fold; 95% CI 46,771–219,146 at week 4), significantly outperforming engineered K562 (4.4-fold greater; 95% CI 1.01 to 18.54; *p* = 0.0479) while maintaining high purity and equivalent cytotoxicity. Substantial inter-donor variability influenced expansion magnitude, though relative feeder performance remained consistent across donors. **Conclusions:** Genetically modified ARH-77 feeder cells provide a potent platform for large-scale ex vivo expansion of functional NK cells.

## Simple Summary

Natural killer (NK) cells are powerful immune cells that naturally attack cancer. Using NK cells from healthy donors as a ready-made (“off-the-shelf”) treatment could be safer and more scalable than personalized therapies that use a patient’s own cells. One challenge is growing sufficient NK cells in the lab from a limited blood sample. Here, we tested a new feeder cell line, ARH-77, and compared it with the commonly used K562 cells, which are known to support NK cell growth. We further engineered both feeder cells with extra “booster” proteins to stimulate even greater growth. Under the best conditions, engineered ARH-77 cells multiplied NK cells more than 100,000-fold in one month. The expanded NK cells remained highly effective at killing cancer targets and stayed mostly pure. Although results varied among donors, ARH-77 worked efficiently to grow NK cells from peripheral blood samples. This new method could help make reliable, large batches of NK cells for better cancer treatments.

## 1. Introduction

Adoptive cell therapy (ACT) of allogeneic natural killer (NK) cells has emerged as a promising immunotherapy for cancer. Allogeneic NK cells can be administered without causing graft-versus-host disease (GvHD) or severe cytokine release syndrome, key safety concerns associated with T cell therapies. Therefore, unlike autologous T cell-based therapies, which require patient-specific manufacturing, allogeneic NK cells can be sourced from healthy donors, expanded ex vivo, and cryopreserved, thereby creating an “off-the-shelf” product readily available for infusion. Clinical trials thus far have demonstrated both the safety and anti-tumor activity of adoptively transferred NK cells, particularly in patients receiving chimeric antigen receptor (CAR)-expressing NK cells [1,2,3,4,5]. Achieving therapeutically relevant numbers of NK cells represents a key challenge in the cell therapy manufacturing pipeline. NK cells constitute approximately 5–20% of adult peripheral blood mononuclear cells, whereas umbilical cord blood is relatively enriched in NK cells compared with adult peripheral blood. Therefore, extensive ex vivo expansion is required to generate sufficient cell numbers for therapeutic applications [6]. Various methodologies have been explored to optimize NK cell expansion, with differences in starting cell source, pre-expansion sorting strategies, culture conditions, and stimulation protocols. Refining these approaches is crucial for enhancing NK cell yield and potency, ultimately enabling the development of scalable and effective NK cell therapies.

Ex vivo activation of NK cells with interleukin (IL)-2 has long been a cornerstone of NK cell immunotherapies [7]. While IL-2 supports NK cell survival and stimulates their cytotoxic and proliferative pathways, it drives only limited expansion [8,9]. For instance, Fujisaki et al. expanded isolated NK cells 4-fold in IL-2 over a week, and Granzin et al. achieved 14-fold over 2 weeks [8,9]. Similarly, IL-15, which shares receptor signaling subunits with IL-2, can also sustain and stimulate NK cells [7]. This led to its transgenic expression in NK cells to improve persistence post-ACT [10,11]. However, NK cells cultured with exogenous IL-15 or engineered to express membrane-bound (mb) IL-15 failed to exhibit robust expansion ex vivo [8,10,12,13]. Beyond IL-2 and IL-15, other cytokines have been investigated for NK cell activation and expansion, including IL-21 and the combination of IL-12, IL-18, and IL-15, which induces a “cytokine-induced memory” NK cell phenotype with enhanced functional properties [13,14]. In general, while methods using cytokines alone can generate NK cells with advantageous phenotypes, they typically fail to drive robust expansion and, therefore, limit the scale of doses able to be administered in the clinic.

Feeder cell-stimulated expansion, in which NK cells are co-cultured with irradiated transformed cells, has significantly advanced NK cell dose manufacturing, enabling expansion levels far beyond what has been possible with cytokines alone. As early as the late 1980s, studies demonstrated that NK cells proliferate rapidly when co-cultured with Epstein–Barr virus (EBV)-transformed lymphoblastoid cells in the presence of IL-2 [15,16]. Today, the HLA-I-deficient K562 cell line, originally derived in 1975 from a patient with myelogenous leukemia, has become the foundation for feeder cell engineering due to its high sensitivity to NK cell-mediated lysis [17]. Importantly, feeder cells are lethally irradiated in advance of co-culture to prevent their proliferation. Furthermore, feeder cells are typically co-cultured with NK cells in media supplemented with IL-2 or IL-15 [7].

A pivotal breakthrough came in 2005, when K562 cells were engineered to express mbIL-15 and CD137L (4-1BBL), resulting in over 1000-fold expansion of NK cells from PBMCs in three weeks [18]. This finding catalyzed further efforts to optimize feeder cell-based NK expansion. Later, K562 cells were engineered to express CD137L and mbIL-21 [19]. Remarkably, this engineered feeder cell line expanded peripheral blood NK cells nearly 50,000-fold in 3 weeks. More recently, feeder cells engineered to express mbIL-21, CD137L, and CD48 have been integral to CD19 CAR NK cell clinical trials, facilitating the expansion of cord blood-derived NK cells to clinically relevant numbers [20]. Advancements in feeder cell engineering have made it possible to administer doses of hundreds of millions, and even billions, of NK cells to patients in clinical trials [3,21,22]. Since NK cells express a variety of activating receptors and can be stimulated to kill a variety of cell lines, ongoing research continues to test different combinations of ligands and cytokines across various cell lines to further enhance NK cell expansion [23,24,25,26,27].

Here, we utilized the ARH-77 cell line, a B lymphoblastic cell line originally isolated from a patient with plasma cell leukemia, as a feeder cell capable of inducing NK cell expansion in excess of unmodified K562 cells [28]. ARH-77 cells are EBV-transformed, testing positive by PCR for the EBV genome but negative for EBV proteins and thus have been categorized as latently infected without production of active viruses [29]. Our foundational finding was that unmodified ARH-77 cells expanded NK cells to greater numbers than the widely used K562 cell line. This result prompted us to engineer ARH-77 cells to express B7-H6, CD137L, IL-15, and IL-15R⍺, with the goal of further enhancing their capacity to drive NK cell expansion. CD137L was selected as it has previously been shown to drive extensive NK cell expansion when expressed on feeder cells. Expression of IL-15 and IL-15R⍺ is intended to provide NK cells with the vital IL-15 trans-presentation signal that supports their survival, cytotoxicity, and proliferation. B7-H6, the ligand for the activating receptor NKp30, was included to provide an additional stimulatory signal through a distinct innate immune axis. Given their ability to robustly expand primary NK cells while preserving cytotoxic function, the genetically modified ARH-77 cells represent a promising feeder cell platform for clinical NK cell applications.

## 2. Materials and Methods

### 2.1. Cells and Culture

The human plasma cell leukemia cell line ARH-77 and the human myelogenous leukemia cell line K562 cells were obtained from the American Type Culture Collection (ATCC, Manassas, VA, USA) and cultured in complete RPMI-1640 medium supplemented with 10% fetal bovine serum (FBS), 100 U/mL of penicillin, and 100 µg/mL of streptomycin (All from WELGENE, Gyeongsan, Republic of Korea). The human embryonic kidney cell line HEK293T cells were obtained from Takara and cultured in complete DMEM high glucose (WELGENE) supplemented with 10% FBS, 100 U/mL of penicillin, and 100 µg/mL of streptomycin.

### 2.2. Generation of Genetically Engineered Feeder Cells Expressing B7-H6, CD137L, IL-15 and IL-15Rα

The cloned B7-H6 cDNA, IL-15 cDNA, IL-15Rα cDNA, and CD137L cDNA were transferred to the lentiviral expression vector pLVX-EF1a-IRES-Puro (Takara Bio Inc., Shiga, Japan) for Lentiviral production. The B7-H6 and EGFP (Enhanced Green Fluorescent Protein) genes were inserted into the multiple cloning sites of the pLVX-EF1a-IRES-Puro plasmid (designated pLVX-EF1a-B7H6/GFP-IRES-Puro [pLVX∼B7H6]). IL-15 and RFP647 (Red Fluorescent Protein 647) genes were respectively inserted into multiple cloning sites of pLVX-EF1a-IRES-Puro plasmid (named pLVX-EF1a-IL15-IRES-RFP647 [pLVX∼IL-15]). Blue Fluorescent Protein (BFP) genes were also encoded in the plasmid expressing IL-15Rα genes (named pLVX-EF1a-IL15Rα/BFP-IRES-Puro [pLVX∼IL-15Rα]). All vectors were verified by DNA sequencing. The lentivirus was produced by co-transfecting HEK293T cells with the respective lentiviral transfer plasmid (10 µg) together with Lenti-X™ Packaging Single shots (Takara). Recombinant lentivirus was harvested 48∼72 h following co-transfection into HEK293T cells cultured in DMEM medium supplemented with 10% FBS. The transfections were performed using Lipofectamine 3000 (Invitrogen, Carlsbad, CA, USA) according to the manufacturer’s instructions. After purification of the virus supernatant, the viral titer was determined using the Lenti-X qRT-PCR Titration Kit (Takara). The genetically modified ARH-77 and K562 cell lines were generated by sequential lentiviral gene transfer of co-stimulatory membrane-bound CD137L, B7-H6, IL-15, and IL-15Rα.

For transduction, ARH-77 and K562 cells were seeded into 24-well plates at 2 × 10^5^ cells/well, respectively, and recombinant lentivirus was added to the wells along with 8 µg/mL polybrene. The mixture was then centrifuged at 1500× *g* at 25 °C for 2 h and incubated at 37 °C in 5% CO_2_. After a 48-h incubation, the virus-containing medium was removed and replaced with 2 mL of fresh culture medium. Transduction efficacy was evaluated daily with an inverted fluorescence microscope. A week after transduction, fluorescence-positive cells were sorted using FACSAria™ III (Becton Dickinson, Franklin Lakes, NJ, USA) and then maintained in RPMI-1640 supplemented with 10% FBS.

### 2.3. Real-Time Reverse Transcription-Polymerase Chain Reaction (RT-PCR)

For the quantitation of mRNA levels that encoded CD137L, B7-H6, IL-15, and IL-15Rα in genetically engineered feeder cells after transduction, quantitative RT-PCR was performed using a QuantiTect SYBR Green PCR Kit and a Rotor-Gene Q (QIAGEN, Hilden, Germany) according to the manufacturer’s instructions. Total RNAs were isolated by RNAiso Plus (Takara), and cDNA was synthesized by ImProm-II™ Reverse Transcription System (Promega, Madison, WI, USA). Primers used for expression analysis were as follows: for β-actin (F; CATCCTCACCCTGAAGTA, R; GAAGGTCTCGAACATGAT), B7H6 (F; ATCCCCGGTATCTGGAGGT, R; AGGTGAAGTTGCGGCTCT) and CD137L (F; GCACACTCGGGAGCTGAT, R; GAGGCAGGTCCAAGGTCA). The thermal cycling conditions were 95 °C for 15 min, followed by 45 cycles at 94 °C for 30 s, 53 °C for 30 s, and 72 °C for 30 s. All samples were tested in triplicate. The relative amount of target gene mRNA was calculated on the basis of its threshold cycle (Ct) compared to the Ct of β-actin. The results are presented as 2^−(Ct of target − Ct of β-actin)in arbitrary units.

### 2.4. Expansion of NK Cells

This study was approved by the Institutional Review Board of Chonnam National University Hwasun Hospital (No. CNUHH-2019-073; approved on 12 April 2019 and 2021-045; approved on 25 November 2021), and no data were used for personal identification of human peripheral blood mononuclear cells (PBMCs). Human PBMCs from healthy donors were isolated by density-gradient centrifugation at 2300 rpm for 25 min using Ficoll-Hypaque (d = 1.077, Lymphoprep; Axis-Shield, Scotland, UK) and washed twice with phosphate-buffered saline (PBS, WELGENE). PBMCs were co-cultured with 100 Gy gamma ray-irradiated feeder cells (ARH-77, K562, genetically modified ARH-77 or genetically modified K562 cells) in 24-well plates with RPMI-1640 complete medium (10% FBS, 100 U/mL penicillin, 100 µg/mL streptomycin, and 4 mmol/L L-glutamine) or DMEM/F-12 complete medium (Thermo Fisher Scientific, Waltham, MA, USA) (10% human AB serum, 10 mL/L Glutamax, 24 μM β-mercaptoethanol, 50 μM Ethanolamine, 20 μg/mL ascorbic acid, 5 ng/mL sodium selenite) containing 10 U/mL rhIL-2 and 5 ng/mL rhIL-21 (PeproTech, Cranbury, NJ, USA). PBMCs were seeded at an initial density of 3 × 10^6^ cells per well in 24-well plates and co-cultured with irradiated feeder cells at a ratio of 6:1. Cultures were monitored every 1–2 days and expanded by splitting into additional wells or transferring to larger culture vessels to maintain optimal cell density and prevent overgrowth. After 1 week, the IL-2 concentration was increased to 100 U/mL, and 5 ng/mL rhIL-15 was added to the medium for 28 days. For each donor, PBMCs were expanded using the same fixed starting number of PBMCs and feeder cells. Feeder cells were added only at culture initiation (day 0), and no additional feeder cells were supplied during the expansion period. Feeder cells were lethally irradiated prior to co-culture and were no longer detectable after approximately six days under the conditions used. The medium was replaced every 2–3 days. Expanded NK cells were harvested on days 14, 21, and 28, and used for further experiments. The absolute number of NK cells was calculated by multiplying the total viable number of cells by the percentage of CD56^+^CD3^−^ cells determined by flow cytometry.

### 2.5. Flow Cytometry Analysis

Fluorescence-activated cell sorting (FACS) analysis was performed using the following monoclonal antibodies (mAbs): anti-CD3 (FITC), anti-CD56 (APC), anti-CD16 (PE), anti-CD335/NKp46 (PE), anti-CD336/NKp44 (PE), anti-CD337/NKp30 (PE), anti-CD226/DNAM-1 (PE), anti-NKG2D (PE), anti-CD96 (PE), anti-CD158b (PE), and anti-ULBP2 (BV421) (all from BD Bioscience, San Jose, CA, USA); anti-ULBP1 (PerCP), anti-ULBP3 (Alexa Fluor 488), and anti-ULBP4 (APC) (all from R&D Systems, Minneapolis, MN, USA); anti-MICA/B (PE-Cy7), anti-HLA-ABC (Super Bright 702), anti-B7-H6 (PE), and anti-IL-15 (APC) (all from Invitrogen); and anti-HLA-E (PE-Cy7), anti-CD137L (APC/Fire 750), anti-CD137L (APC), anti-B7-H6 (APC), and anti-IL-15Rα (APC) (all from BioLegend, San Diego, CA, USA). Isotype controls were run in parallel. Flow cytometry analyses were performed using a FACSCanto II (BD Bioscience). Data were analyzed using FlowJo software (Version 10.4.1, FlowJo LLC, Ashland, OR, USA).

### 2.6. Cytotoxicity Assay

To evaluate cytotoxic function following expansion, NK cell cytotoxicity against K562 target cells (a standardized NK-sensitive reference) was measured using a highly sensitive WST-8-based colorimetric assay (Bimake, Houston, TX, USA) as described previously [30,31]. Briefly, the K562 cells (1 × 10^6^) were placed in a 96-well flat-bottom plate in triplicate and then mixed with expanded NK cells at a 0.25:1 effector to target (E:T) ratio. NK cell purity was quantified by flow cytometry prior to cytotoxicity assays, and effector cell numbers were adjusted to normalize E:T ratios based on absolute NK cell numbers. The plates were centrifuged at 1500 rpm for 3 min and then incubated at 37 °C in a 5% CO_2_ incubator for 3 h. Subsequently, 10 μL WST-8 (Bimake) was added to each well; the plates were incubated again for 1 h. The supernatant was then transferred into new plates. Absorption at 450 nm (A_450_) was measured using the SpectraMax M2 (Molecular Devices, San Jose, CA, USA). The percentage of cytotoxicity was calculated using the following equation: 100% − 100 × [A_450_ of effector cell-treated target cells − A_450_ of effector cell (background of effector cells)]/[A_450_ of target cells − A_450_ of target cells with no WST-8 (background of target cells)].

### 2.7. Statistical Analysis

All experiments were performed in triplicate for each condition unless otherwise stated. All statistical analyses were performed in R (v4.4.0). Fold expansion data were log-transformed and analyzed using linear mixed-effects models implemented via the lme4 package (v1.1-36). This approach was selected for its ability to model repeated measures, account for donor-specific heterogeneity, and appropriately handle unbalanced data arising from occasional missing observations [32,33]. NK cell purity and cytotoxicity, expressed as proportions, were analyzed using generalized linear mixed-effects models assuming a beta distribution with a logit link, implemented via glmmTMB (v1.1.10). Within these models, fixed effects included feeder cell type, time (modeled as an orthogonal second-order polynomial to flexibly capture nonlinear expansion trajectories), and their interaction. Random effects were specified to reflect the hierarchical structure of the experiment: random intercepts and slopes were included for each donor and for each nested donor-by-feeder combination. This structure accounted for differences in NK cell expansion across donors, including inter-individual variability in proliferative kinetics and donor-specific responses to distinct feeder conditions. To justify the inclusion of random effects, we compared the full linear mixed-effects model with nested reduced models and found that the addition of donor:cell-level random effects (intercept and linear slope) significantly improved model fit over a donor-only random-effects model (*p* = 0.002). Furthermore, the complete random effects structure substantially outperformed a fixed-effects-only model (*p* < 0.0001), confirming that donor and donor:cell-specific variability improve models of NK cell expansion trajectories. Pairwise contrasts of estimated marginal means at weeks 2, 3, and 4 were conducted using the emmeans package (v1.10.7), with Tukey-adjusted *p* values to control for multiple testing. In select experiments, non-parametric Friedman tests or Kruskal–Wallis tests were employed to compare multiple groups via rstatix (v0.7.2). An adjusted *p* value < 0.05 was considered statistically significant. Data visualizations were generated using ggplot2 (v3.5.1), ggpubr (v0.6.0) and in BioRender Lee, S. (2026) https://BioRender.com/b3y45bu (accessed on 28 May 2026).

## 3. Results

### 3.1. ARH-77 Cells Demonstrate Enhanced Efficacy in Promoting NK Cell Activation and Proliferation

To identify an efficient feeder cell line for the expansion of primary NK cells, we screened several hematologic malignancy-derived cell lines. In particular, we tested EBV-transformed lines, including ARH-77, Daudi, and Raji, as such lines typically express high levels of co-stimulatory molecules and produce stimulatory cytokines, similar to what has been observed in LCL 721.221 cells [34]. Among these candidates, ARH-77 demonstrated the most potent ability to promote NK cell proliferation, outperforming K562 (*p* = 0.0001) (a commonly used feeder line for NK cell expansion) while maintaining comparable NK cell purity after 14 days of expansion (Figure S1).

**Figure 1:**
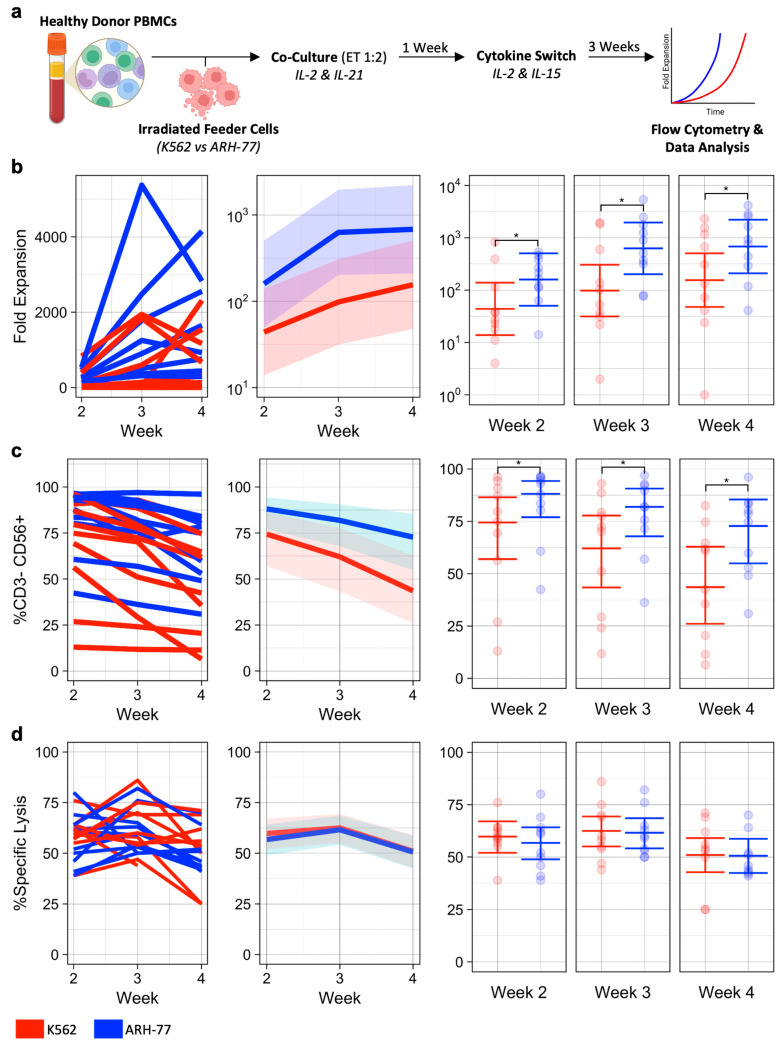
Comparison of NK cell stimulation with unmodified ARH-77 and K562 cells. (**a**) PBMCs (*n* = 10 donors) were cultured with either K562- (red) or ARH-77 (blue)-irradiated feeder cells for four weeks. (**b**) Fold expansion of CD3^−^CD56^+^ NK cells over time, modeled using a linear mixed-effects model. (**c**) NK cell purity (% CD3^−^CD56^+^) and (**d**) NK cell cytotoxicity against K562 targets (0.25:1 E:T ratio) were analyzed using generalized linear mixed models assuming a beta distribution with a logit link. Left panels show individual donor trajectories, middle panels show model-predicted means with 95% confidence intervals and right panels display model-estimated marginal means ± 95% CI overlaid on raw donor-level values. Adjusted *p* < 0.05 denoted by *.

To directly compare the feeder cell potential of ARH-77 to that of K562, we co-cultured PBMCs from healthy donors with the irradiated cell lines in cytokine-supplemented RPMI-1640 complete medium (Figure 1a). Cultures were monitored over four weeks, and NK cell expansion, purity, and cytotoxic function were assessed at weeks 2, 3, and 4. Our linear mixed effects model confirmed a significant effect of feeder cell type on NK cell expansion (*p* = 0.0013) (Figure 1b). Pairwise comparisons revealed that ARH-77 consistently produced greater expansion than K562 across time points. By week 4, using ARH-77 feeder cells resulted in, on average, 4.4-fold (95% CI, 2–10) greater NK cell expansion than K562 feeder cells (*p* = 0.0018). In some cultures, a decrease in cell number between weeks 3 and 4 was observed. This likely reflects a contraction phase following peak expansion rather than overgrowth-related cell death. The model-estimated mean expansion reached 681-fold (95% CI: 206–2258) with ARH-77 compared to 155-fold (95% CI: 47–514) with K562 (*p* = 0.0018). Despite the pronounced differences in cumulative fold expansion, the modeled rate of expansion from weeks 2 to 4 did not differ significantly between feeder cell types, suggesting that an early proliferative advantage prior to the week 2 measurement likely accounts for the divergence in yield (Figure S2A).

The linear mixed-effects model revealed substantial inter-donor variability in NK cell expansion kinetics, which dominated over residual error (residual SD = 0.23 log_10_ units) (Figure S2B). Inter-donor differences were the most pronounced variance in expansion trajectory (donor-level random intercept SD = 0.63 log_10_ units; donor-level random linear slope SD = 1.28 log_10_ units). This heterogeneity likely reflects intrinsic donor differences, including varying baseline NK cell frequencies in PBMCs and innate NK proliferative potential. The variability was reflected in the wide range of expansions observed at week 4 with ARH-77, spanning from 41-fold to 4151-fold across donors (approximately 100-fold difference). Donor-specific responses to feeder cell type were captured by variation in the linear growth rate between feeder cells within donors (SD = 0.54 log_10_ units), highlighting inter-individual differences in the relative growth advantage conferred by ARH-77 versus K562. Together, these findings demonstrate that while ARH-77 consistently outperformed K562 on average each week (3.6–6.4-fold greater expansion), the magnitude of its superiority was donor-dependent, underscoring the importance of both feeder cell selection and donor characteristics in determining NK cell expansion efficiency.

NK cell purity, defined as the model-estimated proportion of CD3^−^CD56^+^ cells in culture, was significantly higher in ARH-77-stimulated cultures compared to K562 at all assessed time points (Figure 1c). At week 2, ARH-77 co-cultures yielded 88% purity (95% CI: 77–94%) compared to 74% (95% CI: 57–87%) with K562 feeders. This difference widened progressively through week 4, where ARH-77 cultures retained higher purity at 73% (95% CI: 55–85%) versus 43.6% (95% CI: 26–63%) in K562 conditions (*p* < 0.0001). Overall, we observed a decrease in the proportion of CD3^−^CD56^+^ cells between weeks 2 and 4 in both feeder cell conditions. Notably, this was accompanied by an increase in the proportion of CD3^+^CD56^+^ cells, and not CD3^+^CD56^−^ cells (Figure S3). Then, to assess functional competency, we measured the cytotoxic activity of expanded NK cells against K562 targets at an effector-to-target ratio of 0.25:1. Model-estimated percent specific lysis did not differ significantly between conditions, indicating that NK cells expanded with ARH-77 feeder cells retained comparable cytotoxic function to those expanded with K562 (Figure 1d).

**Figure 2:**
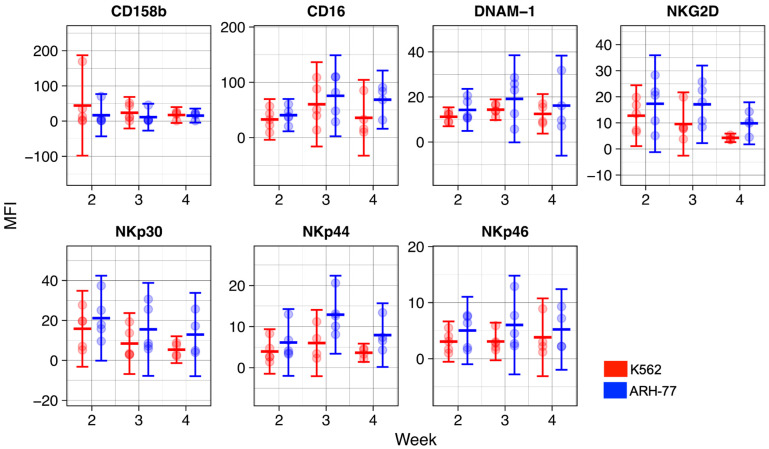
Comparison of NK cell phenotype following expansion with unmodified ARH-77 and K562 cells. PBMCs (*n* = 5 donors) were cultured with either K562 (red) or ARH-77 (blue) feeder cells for four weeks to assess NK cell expansion. At weeks 2, 3 and 4, surface expressions of various receptors central to NK cell function were assessed via flow cytometry. Expression levels of each receptor represent relative mean fluorescence intensity (MFI). Each data point represents an independent donor, and bars represent mean ± SD. Paired Wilcoxon tests were used to compare MFI between feeder conditions at each time point.

To determine whether feeder cell type influences NK cell phenotype, we profiled the expression of key activating and inhibitory surface receptors on NK cells expanded with ARH-77 or K562 feeder cells. Expression levels of CD16, CD158b, DNAM-1, NKG2D, NKp30, NKp44, and NKp46 were assessed at weeks 2, 3, and 4 by flow cytometry (Figure 2). Across all time points, expression of these receptors was largely comparable between conditions, with no statistically significant differences observed. Collectively, these data demonstrate that unmodified ARH-77 feeder cells consistently and more effectively support NK cell expansion and enrichment than K562, while maintaining a comparable cytotoxicity and surface receptor expression. Based on these findings, K562 and ARH-77 were selected for parallel genetic engineering and subsequent comparative analysis.

### 3.2. Generation of Genetically Engineered ARH-77 and K562 Cells

**Figure 3:**
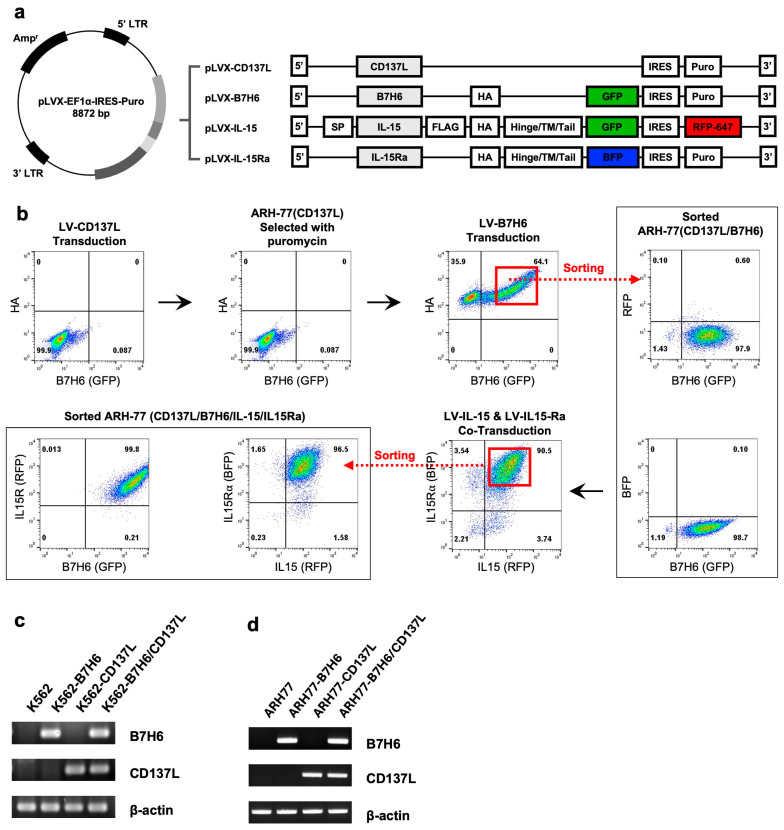
Generation of ARH77-CD137L-B7H6-IL15-IL15Rα feeder cells. (**a**) Schematic representation of CD137L, B7-H6, IL-15 and IL15Rα coding sequences in the lentiviral vector backbone pLVX-EF1a-IRES-Puro. (**b**) The genetically modified ARH-77 and K562 cell lines were generated by sequential lentiviral gene transfer of co-stimulatory membrane-bound CD137L, B7-H6, IL-15 and IL-15Rα. (**c**) mRNA expression of CD137L and B7-H6 in K562, and genetically modified K562 cells, and (**d**) ARH-77, and genetically modified ARH-77 was determined by RT-PCR with β-actin serving as control. Signal peptide (SP); Transmembrane (TM); Hemagglutinin Tag (HA); Internal Ribosome Entry Site (IRES), Puromycin (Puro). Uncropped gel images are available in Supplemental Figure S10.

To enhance the stimulatory capacity of feeder cells, both ARH-77 and K562 lines were genetically engineered to co-express four molecules selected to engage multiple NK cell activation pathways: the 4-1BB ligand CD137L, to deliver a co-stimulatory proliferative signal; B7-H6, a ligand for the activating receptor NKp30, to promote NK cell activation through natural cytotoxicity receptor engagement; and a membrane-bound form of IL-15 (mbIL-15) co-expressed with the IL-15 receptor α-chain (IL-15Rα), to provide a physiologically relevant trans-presentation signal that supports NK cell proliferation, activation, and survival more effectively than IL-15 alone. Each ligand was introduced into the parental cell lines by sequential lentiviral transduction (Figure 3a).

First, both ARH-77 and K562 cells were transduced to express CD137L and selected with puromycin, followed by a second transduction to force B7-H6 expression (Figure 3b). FACS of GFP^+^ cells selected the transduced population and quantitative PCR confirmed successful transgene expression, with transduced cells exhibiting elevated mRNA levels of CD137L and B7-H6 relative to unmodified parental lines (Figure 3c,d).

At this stage, an experiment was conducted to compare the stimulatory nature of K562 cells overexpressing either CD137L or B7-H6. For this purpose, PBMCs were co-cultured with either unmodified K562 cells, B7-H6-transduced K562 (K562-B7H6) cells, or CD137L-transduced K562 (K562-CD137L) cells in RPMI supplemented with cytokines (Figure S4A). At week 2, the model-estimated fold expansion of CD3^−^CD56^+^ NK cells was comparable across all groups. By week 3, both K562-B7H6 (*p* = 0.0004) and K562-CD137L (*p* < 0.0001) significantly outperformed unmodified K562. Additionally, K562-CD137L supported significantly greater expansion than K562-B7H6 (*p* = 0.0016). These effects persisted at week 4, with mean fold expansions using K562-B7H6 (*p* < 0.0001) and K562-CD137L (*p* < 0.0001) being greater than unmodified K562 and K562-CD137L stimulation expanding NK cells greater than K562-B7H6 (*p* = 0.0014). To examine kinetics, model-estimated rates of change were compared at each time point (Figure S4B). At week 2, K562-CD137L-stimulated cultures exhibited a significantly steeper expansion trend compared to unmodified K562 (*p* = 0.0026). By week 3, both K562-CD137L and K562-B7H6 drove significantly greater expansion than K562 (*p* < 0.0001 and *p* = 0.0004, respectively), and superior expansion in the CD137L condition compared to B7-H6 was observed (*p* = 0.0483). These differences diminished by week 4, suggesting that the effect of co-stimulation on expansion kinetics was most pronounced during early culture. These findings suggest that overexpression of B7-H6 or CD137L enhances NK cell expansion compared to unmodified K562, with CD137L providing the strongest overall support, particularly in early culture phases.

The proportion of CD3^−^ CD56^+^ cells expanded from PBMCs with each feeder cell was largely comparable, with purities above 90% (Figure S4C). While there were statistically significant differences in proportion, the magnitudes of those changes were small. Finally, cytotoxicity against K562 target cells was assessed across different feeder cell conditions throughout NK cell expansion (Figure S4D). NK cells expanded with K562-CD137L or K562-B7H6 exhibited significantly greater cytotoxicity at week 2 (*p* = 0.0007 and <0.0001, respectively) and week 3 (*p* = 0.0134 and 0.0069, respectively) compared to K562. However, by week 4, cytotoxicity in the B7-H6 group declined markedly, with percent specific lysis dropping from 85% at week 2 to 58% at week 4, becoming indistinguishable from unmodified K562 (*p* = 0.742). In contrast, K562-CD137L-expanded NK cells maintained superior cytotoxicity throughout the culture period and remained significantly more cytotoxic than those expanded with K562 at week 4 (*p* = 0.0012). Comparison of the model-estimated rate of change in cytotoxicity further revealed a significantly greater decline in B7H6-expanded NK cells than in both the CD137L and K562 groups when calculated at week 3 (*p* = 0.0008 and 0.001, respectively) (Figure S4E). These results indicate that while both co-stimulatory modifications initially enhance NK cell function, CD137L overexpression provides more sustained cytotoxic potency over prolonged culture, consistent with its previously observed superior effects on expansion kinetics.

Following the verification of NK cell stimulation by B7-H6 as described above, mbIL-15 and IL-15RA genes were co-transduced into ARH-77 and K562 cells (Figure 3b). FACS of RFP+ BFP+ cells selected the transduced population. Ultimately, this process led to the construction of genetically modified ARH77-CD137L-B7H6-IL15-IL15Rα and K562-CD137L-B7H6-IL15-IL15Rα feeder cells. The purity of the desired feeder cells after sorting exceeded 96% (Figure 3b). The expression of all transduced genes in ARH77-CD137L-B7H6-IL15-IL15Rα cells and K562-CD137L-B7H6-IL15-IL15Rα cells was observed to remain stable for a duration of at least one year. Flow cytometry confirmed that irradiation (100 Gy) did not result in substantial changes in the surface expression of ligands engineered for expression in ARH77-CD137L-B7H6-IL15-IL15Rα (Figure S5A). As expected, irradiation altered the expression of other known radiation-induced NK cell ligands, including ULBP-1, ULBP-2, MIC-A, and HLA-E, on parental ARH-77 and K562 cells (Figure S5B). These data indicate that irradiation does not significantly alter the expression of key stimulatory ligands in either parental or engineered feeder cells.

### 3.3. Genetically Engineered ARH-77 Feeder Cells Induced Robust Expansion of NK Cells from PBMCs

**Figure 4:**
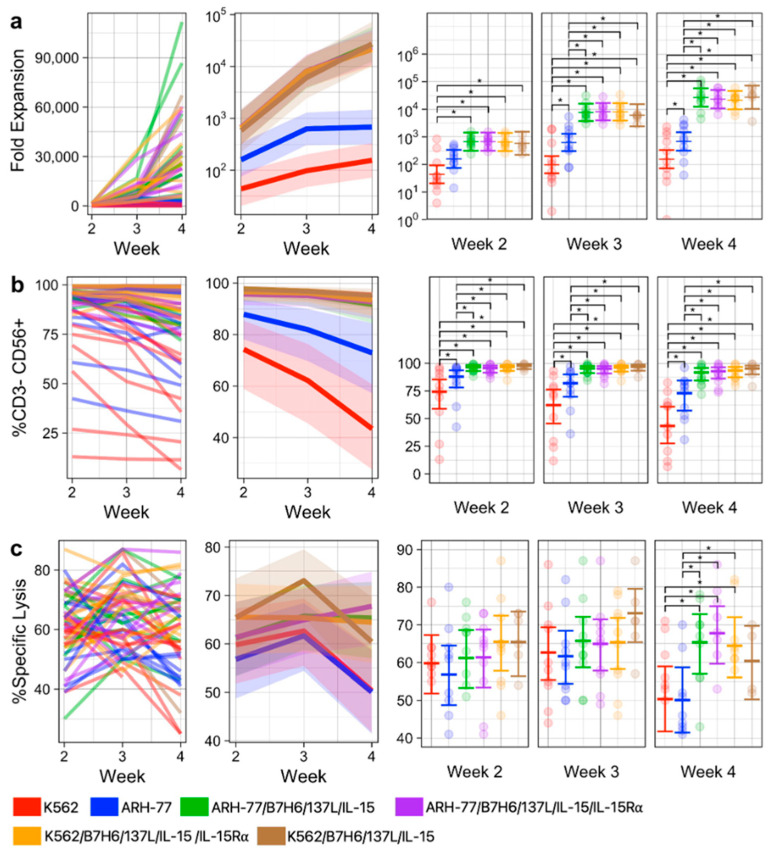
NK Cell Expansion with Modified Feeder Cells In RPMI. PBMCs (*n* = 10 donors) from healthy donors were cultured with various genetically engineered feeder cells to assess their effects on NK cell expansion and function. (**a**) Fold expansion of CD3^−^CD56^+^ NK cells over time, modeled using a linear mixed-effects model. (**b**) NK cell purity (% CD3^−^CD56^+^) and (**c**) NK cell cytotoxicity against K562 targets (0.25:1 E:T ratio) were analyzed using generalized mixed models assuming a beta distribution with a logit link. Left panels show individual donor trajectories, middle panels show model-predicted means with 95% confidence intervals, and right panels display model-estimated marginal means ± 95% CI overlaid on raw donor-level values. Statistical significance was determined by pairwise comparisons of model-estimated means, adjusted using the Tukey correction. Adjusted *p* < 0.05 is indicated by *. Due to substantial inter-donor variability, individual donor trajectories are shown to illustrate the range of individual responses, while model-predicted trends are provided to summarize average feeder cell expansion.

To assess the impact of genetic modification on feeder cell efficacy, we co-cultured PBMCs from healthy donors with various engineered and irradiated K562 and ARH-77 feeder cell lines in cytokine-supplemented RPMI complete medium and quantified NK cell expansion over a 4-week period (Figure 4a). This analysis was performed in RPMI to determine whether genetic engineering itself enhanced feeder cell performance relative to the corresponding unmodified parental lines under the baseline culture condition used in the study. By week 4, ARH77-CD137L-B7H6-IL15 and ARH77-CD137L-B7H6-IL15-IL15Rα feeder cells drove model-estimated mean fold expansions of 26,705 (95% CI: 12,408–57,475) and 23,243 (95% CI: 10,800–50,025), respectively. Similarly, K562-CD137L-B7H6-IL15 and K562-CD137L-B7H6-IL15-IL15Rα feeder cells supported expansions of 27,217 (95% CI: 10,258–72,208) and 21,336 (95% CI: 9913–45,920). In contrast, unmodified K562 and ARH-77 feeder cells resulted in significantly lower expansion, with model-estimated means of 155 (95% CI: 72–333) and 681 (95% CI: 316–1466), respectively. Pairwise model-based comparisons confirmed that modified feeder cell lines improved NK cell expansion compared to their unmodified controls (*p* < 0.0001). However, there were no significant differences in NK cell expansion between the modified feeder cell lines. Therefore, even though unmodified ARH-77 cells outperformed unmodified K562 in supporting NK cell expansion, the modified ARH-77 and K562 cell lines exhibited comparable performance in these conditions.

In addition to expansion, NK cell purity (proportion of CD3^−^CD56^+^ cells) was increased in all modified feeder cell conditions compared to unmodified controls (*p* < 0.0015) (Figure 4b). NK cell purity levels were comparable across cultures expanded with the modified feeder cell lines. NK cell cytotoxicity, assessed as percent specific lysis against K562 target cells, was similar across all groups at weeks 2 and 3 (Figure 4c). By week 4, however, NK cells expanded with ARH77-CD137L-B7H6-IL15 and ARH77-CD137L-B7H6-IL15-IL15Rα feeder cells exhibited significantly higher cytotoxicity than those expanded with unmodified ARH-77 or K562 (*p* < 0.0056). A similar increase was observed with K562-CD137L-B7H6-IL15-IL15Rα (*p* = 0.0070), while K562-CD137L-B7H6-IL15 did not significantly outperform the unmodified conditions.

Together, these findings demonstrate that the addition of B7H6, CD137L, IL-15, and IL-15Rα to feeder cells substantially enhances their ability to drive NK cell expansion relative to unmodified counterparts. However, no significant differences were observed between the modified K562 and ARH-77 feeder cell lines, indicating that the engineered stimulatory components, rather than the feeder cell background, primarily accounted for the improved expansion under these conditions.

### 3.4. DMEM/F-12 Culture Further Enhances NK Cell Expansion Driven by Genetically Engineered ARH-77 Feeder Cells

**Figure 5:**
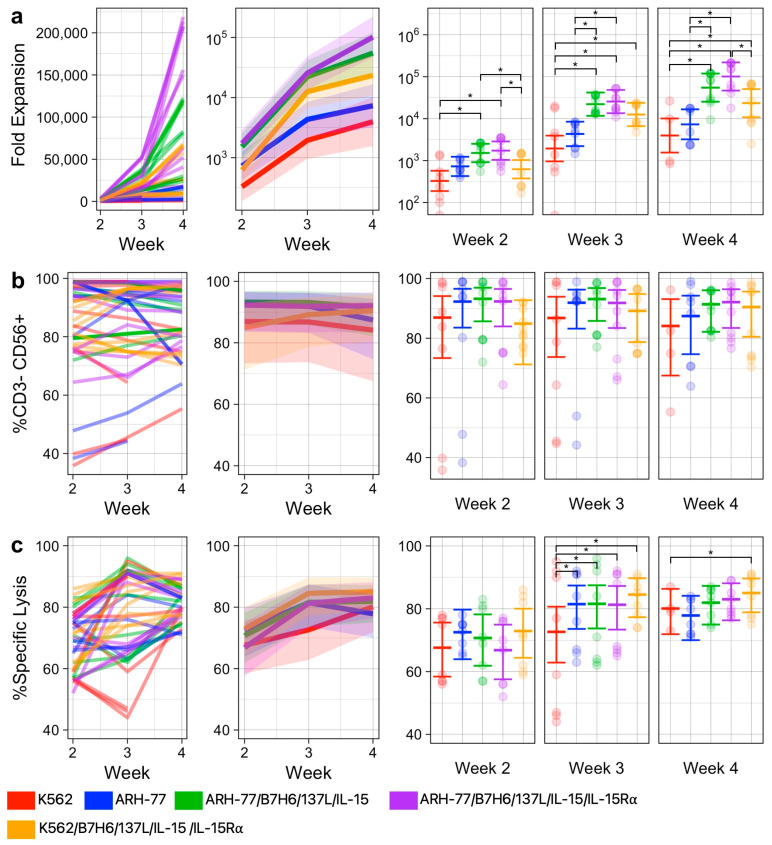
NK Cell Expansion with Modified Feeder Cells In DMEM/F-12. PBMCs (*n* = 10 donors) from healthy donors were cultured with various genetically engineered feeder cells to assess their effects on NK cell expansion and function. (**a**) Fold expansion of CD3^−^CD56^+^ NK cells over time, modeled using a linear mixed-effects model. (**b**) NK cell purity (% CD3^−^CD56^+^) and (**c**) NK cell cytotoxicity against K562 targets (0.25:1 E:T ratio) were analyzed using generalized mixed models assuming a beta distribution with a logit link. Left panels show individual donor trajectories, middle panels show model-predicted means with 95% confidence intervals, and right panels display model-estimated marginal means ± 95% CI overlaid on raw donor-level values. Statistical significance was determined by pairwise comparisons of model-estimated means, adjusted using the Tukey correction. Adjusted *p* < 0.05 is indicated by *.

Prior studies have shown that DMEM/F-12 more effectively supports NK cell expansion than RPMI, prompting us to perform subsequent experiments using DMEM/F-12 as the basal culture medium [35,36]. This section was intended to assess the behavior of the engineered feeder systems under a more optimized expansion context, rather than to serve as a comprehensive head-to-head comparison of alternative NK-cell media. Based on preliminary comparisons among K562-derived feeder cell lines, K562-CD137L-B7H6-IL15-IL15Rα was selected as the reference control for final comparisons with engineered ARH-77 feeder cells (Figure S6A). Under these optimized conditions, ARH-77-derived feeder cells expressing B7-H6, CD137L, and IL-15, either with or without IL-15Rα, supported the highest levels of NK cell expansion over the 4-week culture period (Figure 5a and Figure S6B). By week 4, ARH77-CD137L-B7H6-IL15-IL15Rα feeder cells exhibited the highest expansion overall, with a model-estimated mean of 101,241-fold (95% CI: 46,771–219,146). ARH77-CD137L-B7H6-IL15 also achieved robust expansion (54,922-fold, 95% CI: 25,372–118,885), which was not statistically different from the expansion achieved by ARH77-CD137L-B7H6-IL15-IL15Rα feeder cells. Both genetically modified ARH-77 feeder lines supported significantly greater expansion than unmodified controls (*p* < 0.0041). The ARH77-CD137L-B7H6-IL15-IL15Rα feeder cell line also outperformed K562-CD137L-B7H6-IL15-IL15Rα, driving on average 4.4-fold (95% CI 1.01 to 18.54) greater expansion (*p* = 0.0479). To further compare expansion dynamics, we evaluated the modeled rate of NK cell expansion. At week 3, all modified feeder cell lines drove significantly higher expansion rates compared to unmodified controls (*p* < 0.05) (Figure S6C). However, no significant differences in NK cell expansion rates were detected between the modified feeder cell lines at that time point.

Assessing the random effects variance in the linear mixed-effects model, we observed inter-donor variability in overall expansion rates (donor-level random linear time slope SD = 0.51 log_10_ units; random quadratic time slope SD = 0.97 log_10_ units) (Figure S6D). This heterogeneity resulted in NK cell expansions at week 4 varying from 17,806-fold to 218,853-fold across donors for the best-performing feeder cell line, ARH77-CD137L-B7H6-IL15-IL15Rα (approximately 12-fold range). This variability likely reflects intrinsic donor differences in NK cell proliferative capacity and baseline NK cell frequency in PBMCs. Notably, this donor-level heterogeneity in proliferation kinetics far exceeded the residual variance (SD = 0.14 log_10_ units).

In contrast, donor-specific responses to feeder cell lines were comparatively modest (donor–feeder interaction random linear time slope SD = 0.11 log_10_ units). This suggests that while absolute expansion levels varied considerably between donors, the relative ranking of feeder cell performance was largely preserved across the donor population. Consistent with this interpretation, inspection of individual donor trajectories (Figure S6D) revealed that most donors achieved their highest yields with ARH77-CD137L-B7H6-IL15-IL15Rα feeders, though a subset of donors showed superior expansion with the ARH77-CD137L-B7H6-IL15 variant. Furthermore, modified feeder cells tended to outperform unmodified control feeder cells across donors. Together, these findings indicate that while individual PBMC donor characteristics substantially influence the magnitude of NK cell expansion, the relative performance of different modified feeder cells is more generalizable under these culture conditions.

Mean NK cell purity, assessed weekly as the percentage of CD3^−^CD56^+^ cells, was uniformly high across all modified feeder conditions (Figure 5b and Figure S7). No statistically significant differences were observed between groups at any time point, unlike in RPMI, where modified feeder cells drove a greater proportion of CD3^−^ CD56^+^ cell expansion compared to unmodified feeder cells (Figure 4b). Similarly, cytotoxicity was comparable across conditions, except at week 3, when NK cells expanded with unmodified K562 exhibited reduced killing of K562 targets, resulting in several significant pairwise differences (Figure 5c). Therefore, the marked improvement in expansion with genetically modified ARH-77 feeder cells was not associated with any cytotoxicity.

In a separate experiment, we assessed early NK cell expansion following 1 week of co-culture with ARH77-CD137L-B7H6-IL15-IL15Rα in DMEM/F-12. This condition supported a mean expansion of 146 ± 133-fold (Figure S8), highlighting early proliferative activity that precedes the large-scale expansions observed in long-term cultures. Additionally, we assessed the surface expression of various NK cell-related proteins within the first 2 weeks of expansion stimulated by ARH77-CD137L-B7H6-IL15-IL15Rα (Figure S9). Although the experiment was underpowered to detect statistically significant differences, we observed a substantial shift in the proportions of NK cells expressing CD96 and NKp44.

## 4. Discussion

Optimizing feeder cell-mediated expansion of NK cells is key to driving progress towards effective NK cell-based ACT for cancer treatment. The off-the-shelf nature of NK cell therapy holds great potential, but its success depends critically on the ability to efficiently and reproducibly expand NK cells ex vivo from a blood donation. Here, we provide evidence that the plasma cell leukemic cell line ARH-77 can serve as an effective feeder cell platform, inducing potent NK cell stimulation, especially when engineered to express the stimulatory ligands B7-H6, CD137L, IL-15, and IL-15Rα.

Our feeder cell design is intended to provide multi-axis stimulation to NK cells. CD137L (4-1BBL) was included to provide a stimulatory signal that supports NK cell proliferation and sustained cytotoxic activity, consistent with prior studies and our own observations in K562-CD137L feeders (Figure S4). B7-H6 was incorporated because the NKp30–B7-H6 axis has been implicated in NK cell activation and proliferation [37,38]. In our study, expression of B7-H6 alone significantly enhanced NK cell proliferation relative to unmodified K562 controls (Figure S4A), supporting its use as a stimulatory ligand in this system. However, the early cytotoxicity benefit observed with B7-H6 was not maintained at week 4, in contrast to CD137L-K562 feeders, which continued to support superior effector function (Figure S4D). This transient effect is unlikely to limit the utility of B7-H6 in multi-signal feeder designs, in which CD137L co-expression can compensate for pathway-specific attenuation. Finally, IL-15Rα was included to enhance IL-15 signaling through trans-presentation, a mechanism known to support NK cell proliferation and persistence [7].

Among the various genetically modified feeder lines tested, ARH-77 cells engineered to express B7-H6, CD137L, IL-15, and IL-15R drove the highest NK cell expansion observed in the study (Figure 5). In DMEM/F-12 culture medium supplemented with IL-21, IL-2, and IL-15, ARH77-CD137L-B7H6-IL15-IL15Rα feeder cells drove model-estimated mean NK cell expansions of 1724-fold (95% CI: 1039–2861) at 2 weeks, 25,547-fold (95% CI: 13,487–48,387) at 3 weeks, and 101,241-fold (95% CI: 46,771–219,146) at 4 weeks. For context, the CD137L- and IL-15-expressing K562 feeder cells generated by Imai et al. drove a 1000-fold expansion in 3 weeks [18]. mbIL-21 expressing OCI-AML3 feeder cells drove a mean 1000-fold expansion in 3 weeks [26]. Coculturing NK cells with K562-OX40L cells resulted in a 2000-fold expansion in 4 weeks [24]. The widely used K562-41BBL-mbIL21 from Denman et al. drove 47,967 ± 42,230-fold (mean ± SD) expansion in 3 weeks [19]. Feeder cells generated from 721.221 B-lymphoblastoid cells expressing mbIL-21 expanded NK cells from PBMCs around 100,000-fold in 3 weeks [34]. Methodological differences across studies, cytokine combinations, culture media, and the use of isolated NK cells versus bulk PBMCs limit direct comparisons. Rigorous head-to-head evaluations under standardized conditions will be essential to benchmark current and emerging expansion platforms.

Substantial donor variability in NK cell expansion was observed, underscoring the biological heterogeneity inherent among donor PBMC-derived NK cells. Analysis of the random effects from our linear mixed-effects models resolved this variability into two distinct components. The first source of variation, i.e., inter-donor differences, likely reflects PBMC heterogeneity in baseline NK cell frequency and intrinsic NK cell expansion potential. When expanding NK cells from PBMCs, donors with higher NK cell proportions begin with more NK cells, which could contribute to differences in observed fold expansion [39]. Furthermore, among donors, factors such as donor age, genetic background, prior pathogen exposures (e.g., CMV status), and baseline NK cell phenotype may also drive variation in expansion. Substantial inter-donor variability in NK cell fold-expansion has also been observed in other studies [19,24,39]. The second source of variation, which modeled donor-by-feeder cell interactions, captured the extent to which individual donors responded differently to specific feeder cell modifications, which has implications for the generalizability of feeder cell optimization. In DMEM/F-12, donor–feeder interactions were minor, indicating that feeder cell rankings were largely preserved across donors such that most donors achieved maximal expansion with modified ARH-77 feeder cells. This consistency suggests that under these culture conditions, the biological pathways activated by feeder cell modifications (NKp30 engagement via B7-H6, 4-1BB co-stimulation via CD137L, and IL-15 signaling) operate reliably across diverse donor backgrounds. In other words, while absolute expansion levels varied across donors, the relative performance of feeder cell systems remained more consistent, suggesting that feeder-dependent effects are robust despite inherent biological variability. Taken together, this layered variability presents challenges for clinical standardization and highlights the need for future studies to identify predictive biomarkers to guide donor selection in off-the-shelf NK cell manufacturing.

Our expansion strategy was conducted within bulk PBMCs, which reflects a common pre-clinical strategy to assess whether a novel feeder cell selectively expands NK cells from a heterogeneous starting population, as well as a tested clinical starting point for feeder cell expansion [8,18,19,24,39,40,41,42,43]. However, unlike pre-isolating NK cells, this strategy also introduces variability in initial NK cell purity and can permit the expansion of non-NK cell populations. In particular, the presence and expansion of contaminating T cells are recognized concerns in allogeneic settings due to the associated risk of GvHD. Consistent with these limitations, we observed substantial variability in NK cell purity across expansion conditions and donors. Several expansions failed to achieve or maintain high NK cell purity, defined as the proportion of CD3^−^ CD56^+^ cells, with some cultures showing declining purity over time (Figure 1c, Figure 4b and Figure 5b). This variability likely reflects a combination of inter-donor differences and feeder cell-dependent effects. Supporting this interpretation, NK cell expansions performed in RPMI using unmodified feeder cells resulted in lower NK cell purity compared to modified feeder cells (Figure 4b), suggesting that the inclusion of stimulatory ligands preferentially supports CD3^−^ CD56^+^ NK cell expansion as intended. Furthermore, across donors expanded with ARH77-CD137L-B7H6-IL15-IL15Rα in RPMI, purity at week 4 ranged between 74 and 99%, underscoring substantial donor-dependent variability.

Flow cytometric analysis further suggested that reduced NK cell purity was associated with the concurrent expansion of a CD3^+^ CD56^+^ double-positive population (Figures S3 and S7). A similar phenomenon has been reported in a large study of feeder cell-driven NK cell expansions from 16 donor PBMC samples associated with an allogeneic NK cell clinical trial, in which concurrent expansion of CD3^+^ CD56^+^ cells was observed [39]. This population may represent innate-like T cell subsets, such as NKT cells or γδ T cells, which can expand under conditions similar to those for NK cells [44]. While the expansion of non-NK populations is often viewed as an undesirable outcome, the co-expansion of innate-like T cells with antitumor activity could represent a feature, rather than a limitation, of bulk PBMC-based expansion strategies. Determining the identity, functional properties, and in vivo behavior of these populations will therefore be important for interpreting both efficacy and safety. Future studies will be required to assess whether the presence of non-NK populations enhances antitumor responses, increases the risk of GvHD, or both, in allogeneic contexts. In addition, evaluating whether isolating NK cells before or after ARH-77-based expansion improves consistency, purity, or therapeutic performance is an important next step.

Several limitations of the present study warrant discussion. First, our study was designed to evaluate the overall performance of multi-signal engineered feeder cells rather than to dissect the contribution of each individual ligand. We therefore cannot formally isolate the incremental contribution of any single ligand to the observed expansion. Direct head-to-head comparisons of feeder cells expressing defined ligand combinations (e.g., CD137L alone vs. CD137L + B7-H6) will be necessary in future work to formally establish the added value of each ligand. Second, ligand expression on engineered ARH-77 and K562 feeder cells was confirmed by flow cytometry of fluorescent markers but not absolutely quantified at the per-cell level. Differences in protein expression density between K562- and ARH-77-transduced cell lines cannot, therefore, be excluded as a contributor to the observed differences in NK cell expansion and function. Absolute quantification of surface ligand density will be necessary in future work to disentangle the contribution of the cell background from that of engineered ligand expression levels. Third, in DMEM/F-12 conditions, ARH-77 engineered feeders outperformed K562 engineered to express the same ligand. However, the same comparison was not significant when the experiment was performed in RPMI media. The superior expansion observed in DMEM/F-12 should be interpreted in the context of both feeder-cell engineering and medium composition, as this study was not designed as a direct benchmarking comparison across multiple NK-cell culture media. Fourth, although ARH-77/B7H6/CD137L/IL15/IL15R⍺ achieved the greatest expansion, it was not significantly superior to the same feeder cells without IL-15R⍺. The lack of a measurable effect of IL-15Rα under the current conditions may reflect saturation of expansion signals, and future studies using lower PBMC-to-feeder ratios may help to better delineate its contribution.

Beyond these internal limitations, several considerations will determine whether ARH-77-based feeder systems can be translated to clinical NK cell manufacturing. While this study focused on ex vivo expansion, the in vivo persistence and anti-tumor activity of ARH-77-expanded NK cells remain to be established. Cytotoxicity was assessed using K562 cells as a standardized NK-sensitive reference target, which, while widely used in the NK cell field, does not capture broader tumor specificity and may introduce bias in feeder cell comparisons, given that K562 served as both a feeder and a target in this study. Evaluation against additional tumor targets and in vivo models will therefore be essential to establish translational relevance. Finally, factors including feeder cell safety (notably the use of EBV-transformed cell lines), regulatory requirements for feeder inactivation and removal, starting material heterogeneity, and large-scale manufacturing constraints will influence the feasibility and performance of this platform in clinical settings.

## 5. Conclusions

In summary, we demonstrate that the ARH-77 cell line, particularly when genetically engineered to co-express B7-H6, CD137L, IL-15, and IL-15Rα, serves as an effective feeder platform for the ex vivo expansion of primary NK cells from peripheral blood mononuclear cells. Under optimized culture conditions, this system achieved a mean NK cell fold expansion of 101,241 (95% CI: 46,771–219,146) in 4 weeks while preserving purity and cytotoxicity. Although substantial inter-donor variability in expansion magnitude was observed, the relative superiority of modified ARH-77 feeders remained consistent across donors, underscoring its robustness. These findings establish ARH-77 as a promising alternative feeder cell platform that could enhance the scalability, consistency, and potency of allogeneic NK cell manufacturing for clinical adoptive immunotherapy.

## Data Availability

The data and code supporting the results of this study are available on request.

## Supplementary Materials

The following supporting information can be downloaded at: https://www.mdpi.com/article/10.3390/cancers18111833/s1, Figure S1: Preliminary Comparison of EBV Transformed Cell Line Driven NK Cell Expansion; Figure S2: NK Cell Expansion with Unmodified K562 and ARH-77 Feeder Cells; Figure S3: Representative Purity of K562- and ARH-77-Expanded NK Cells; Figure S4: Effect of CD137L and B7-H6 on NK Cell Expansion, Purity and Cytotoxicity; Figure S5: Effect of irradiation on surface ligand expression in feeder cell lines; Figure S6: NK Cell Expansion with Modified Feeder Cells; Figure S7: Representative Purity of NK Cells Expanded with Modified Feeder Cells; Figure S8: Seven Day Expansion with ARH77-CD137L-B7H6-IL15-IL15Rα; Figure S9: Phenotype of NK Cells Expanded with ARH77-CD137L-B7H6-IL15-IL15Rα. Figure S10: Uncropped RT-PCR gel images corresponding to Figure 3c,d.

## Abbreviations

The following abbreviations are used in this manuscript:

NK	Natural Killer
PBMC	Peripheral Blood Mononuclear Cell
ACT	Adoptive Cell Therapy
GvHD	Graft-versus-Host Disease
CAR	Chimeric Antigen Receptor
IL	Interleukin
mb	Membrane bound
EBV	Epstein–Barr Virus
EGFP	Enhanced Green Fluorescent Protein
RFP647	Red Fluorescent Protein 647
BFP	Blue Fluorescent Protein
mAb	Monoclonal Antibody
FACS	Fluorescence-activated cell sorting
FITC	Fluorescein Isothiocyanate
APC	Allophycocyanin
PE	Phycoerythrin
E:T	Effector to target ratio
