# Supplementary material for: Development of Genetically Modified ARH-77 Feeder Cells for Efficient Expansion of Natural Killer Cells with Potent Anti-Tumor Activity
Source: Cancers (Basel). 2026 Jun 3;18(11):1833. doi: 10.3390/cancers18111833 (PMC13257357; doi:10.3390/cancers18111833)
Supplement: Supplementary file 1 [file cancers-18-01833-s001.zip › cancers-4247877-supplementary.pdf]

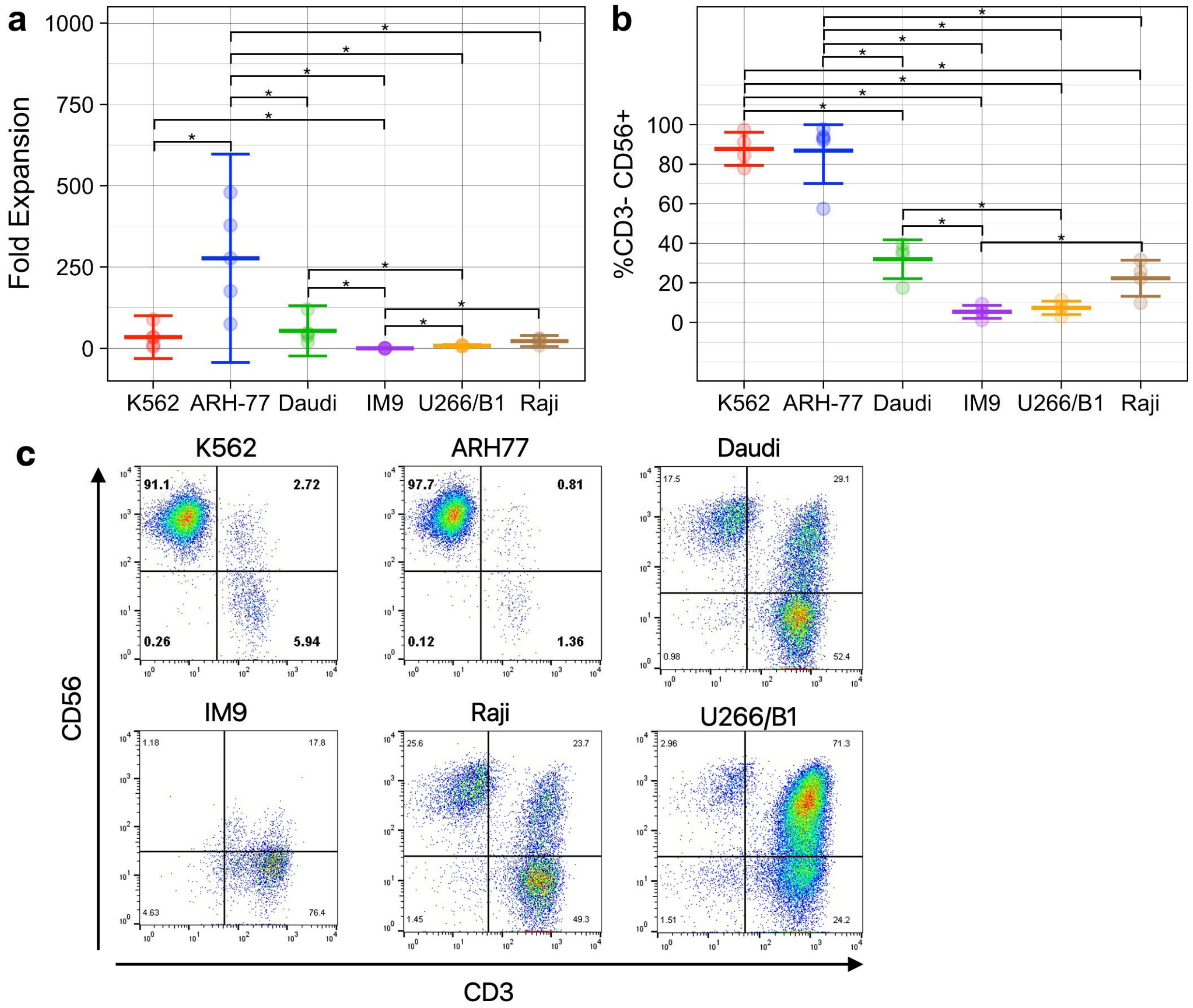

Figure S1. Preliminary Comparison of EBV-Transformed Cell Line-Driven NK Cell Expansion. Cells were expanded from PBCMs (n=5 donors) via co-culture with various irradiated cell lines. On day 14 post-stimulation, (A) fold expansion and (B) NK cell purity (%CD3- CD56+) were quantified. Each data point represents an individual donor; bars denote mean ± SD. Statistical significance was assessed using a linear mixed-effects model for log1p-transformed expansion fold change and a generalized linear mixed-effects model for purity. Pairwise comparisons were performed with Tukey adjustment; Adjusted P-value < 0.05 is indicated by *. (C) Representative flow cytometry plots from (CD3 vs. CD56) illustrating NK cell purity post expansion.

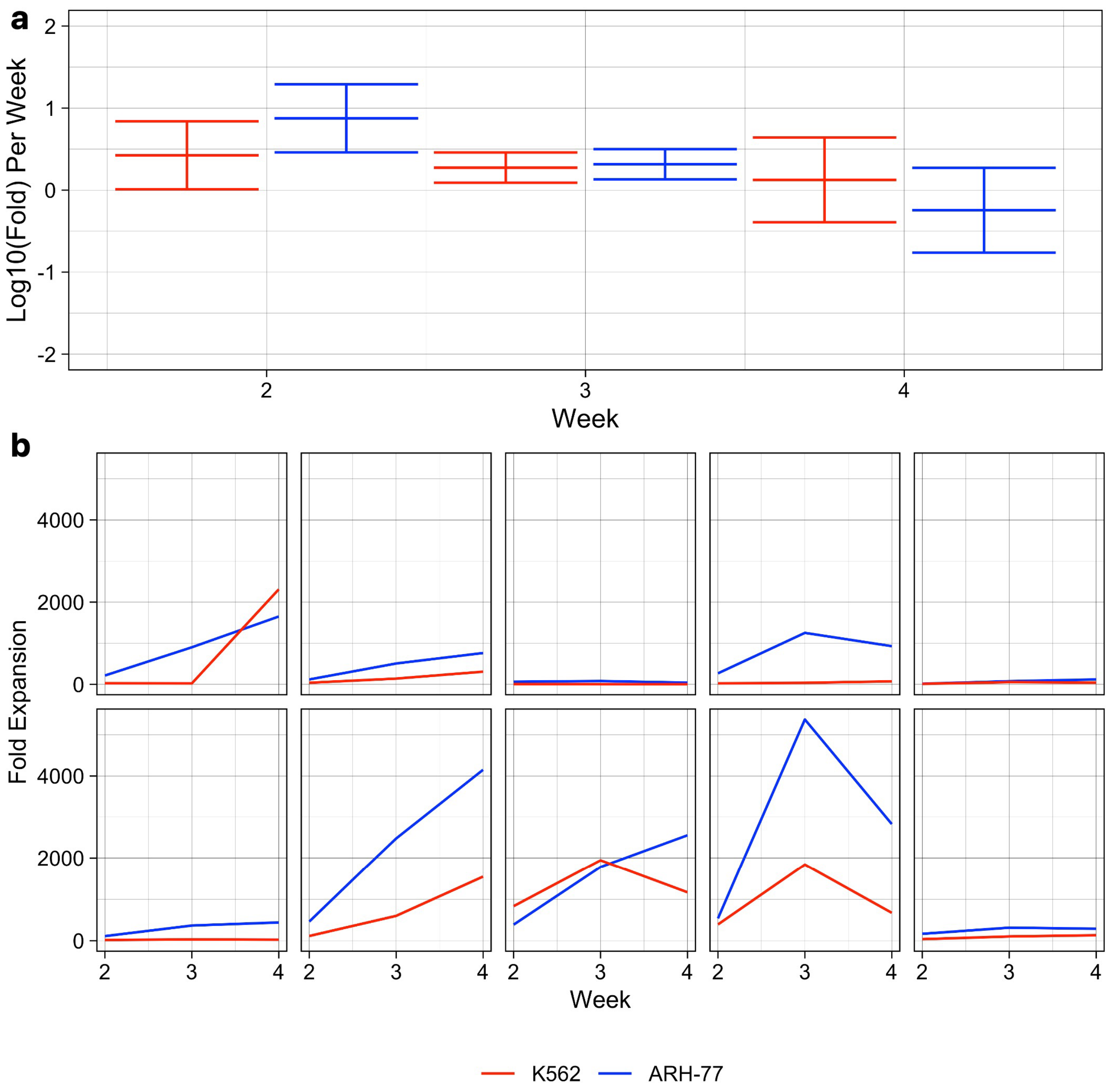

Figure S2. NK Cell Expansion with Unmodified K562 and ARH-77 Feeder Cells. PBMCs (n=10 donors) were cultured with either K562 (red) or ARH-77 (blue) feeder cells for four weeks. Fold expansion was analyzed using a linear mixed-effects model that included a second-order polynomial term for week and random slopes by donor and by donor–feeder combination. (A) The rate of expansion was defined as the slope of $\log_{10}$(fold expansion) with respect to week, estimated at weeks 2, 3, and 4 with 95% CI. (B) NK cell fold expansion over time, with each facet representing a single donor.

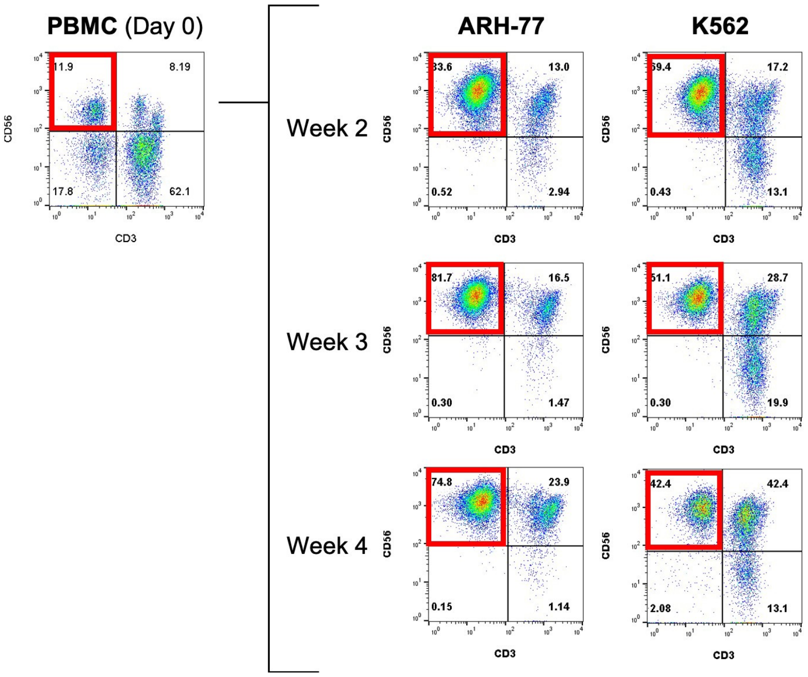

Figure S3. Representative Purity of K562 and ARH-77 Expanded NK Cells. PBMCs from healthy donors (n = 10 donors) were co-cultured with irradiated K562 or AR H-77 feeder cells for four weeks. Representative flow cytometry plots depict the distribution of CD3⁻ CD56⁺ NK cells and other CD3/CD56 expressing populations at the indicated time points.

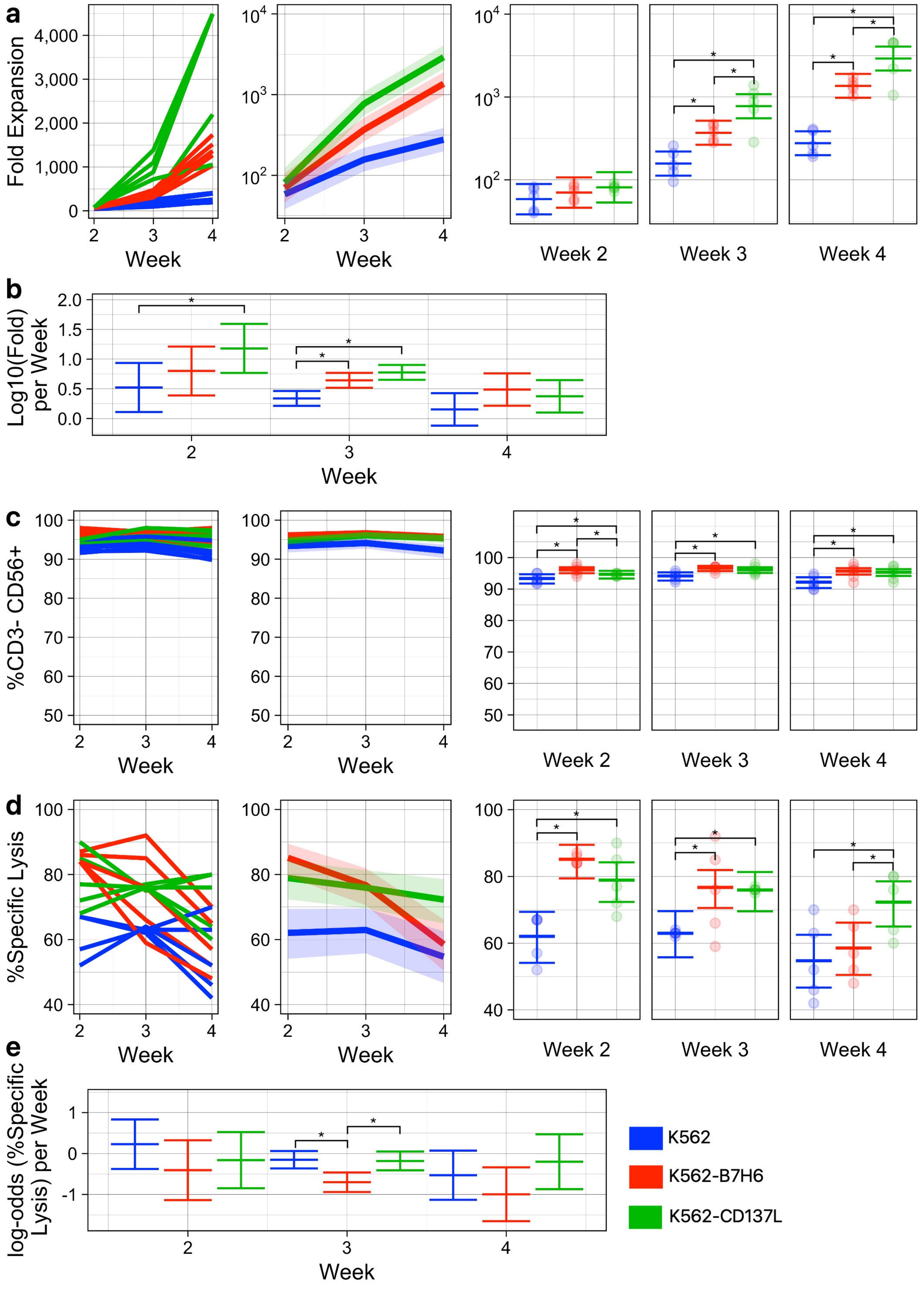

Figure S4. Effect of CD137L and B7-H6 on NK Cell Expansion, Purity and Cytotoxicity. NK cells were expanded from PBCMs (n=5 donors) with either unmodified K562 (blue), K562-B7H6 (red), or K562-CD137L (green) feeder cells. (A) Fold expansion of $CD3^{-}CD56^{+}$ NK cells over time, modeled using a linear mixed-effects model. (B) The rate of expansion was defined as the slope of log_{10}(Fold expansion) with respect to week, estimated at weeks 2, 3, and 4 with 95% CI. (C) NK cell purity (% $CD3^{-}CD56^{+}$) was analyzed using

a generalized mixed model with a beta distribution and a logit link. (D) NK cell cytotoxicity against K562 targets (0.25:1 E:T ratio) assessed using the same GLMM framework. (E) The rate of change in cytotoxic function was defined as the slope of log-odds (% specific lysis) with respect to week. For A, C and E, the left panels show individual donor trajectories, the middle panels show model-predicted means with 95% confidence intervals, and the right panels display model-estimated marginal means ± 95% CI overlaid on raw donor-level values. Statistical significance was determined by pairwise comparisons of model-estimated means, adjusted using Tukey correction. Adjusted $P < 0.05$ is indicated by *.

(A)

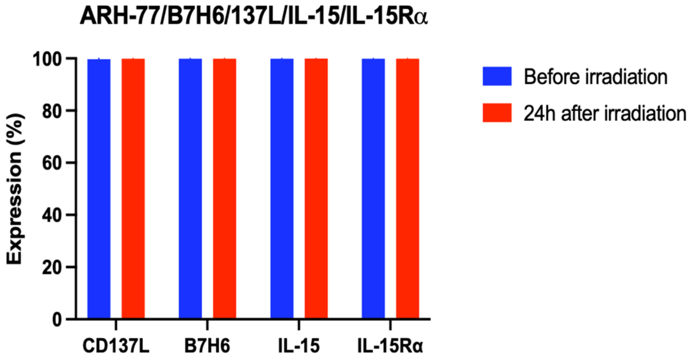

(B)

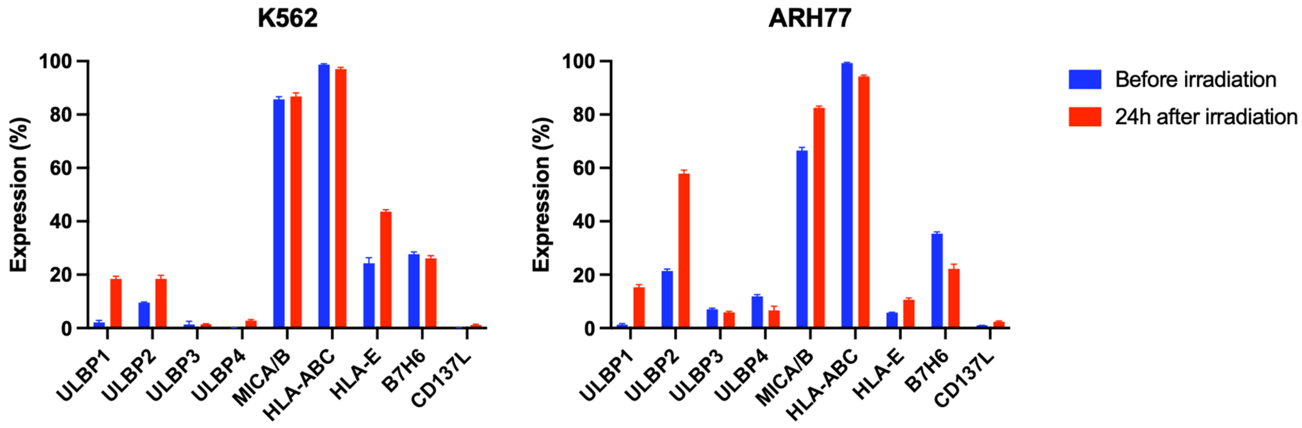

Figure S5. Effect of irradiation on surface ligand expression in feeder cell lines. (A) Flow cytometric analysis of engineered ARH-77 feeder cells expressing B7H6, CD137L, IL-15, and IL-15Rα before and 24 h after irradiation (100 Gy). Expression levels of all engineered ligands were maintained following irradiation. (B) Flow cytometric analysis of surface expression of NK cell-relevant ligands, including NKG2D ligands (ULBP1-4, MICA/B), B7H6, HLA-ABC, HLA-E, and CD137L, in K562 and ARH-77 cells before and 24 h after irradiation (100 Gy). Representative data from independent experiments are shown. These data indicate that irradiation does not significantly alter the expression of key stimulatory ligands in either parental or engineered feeder cells.

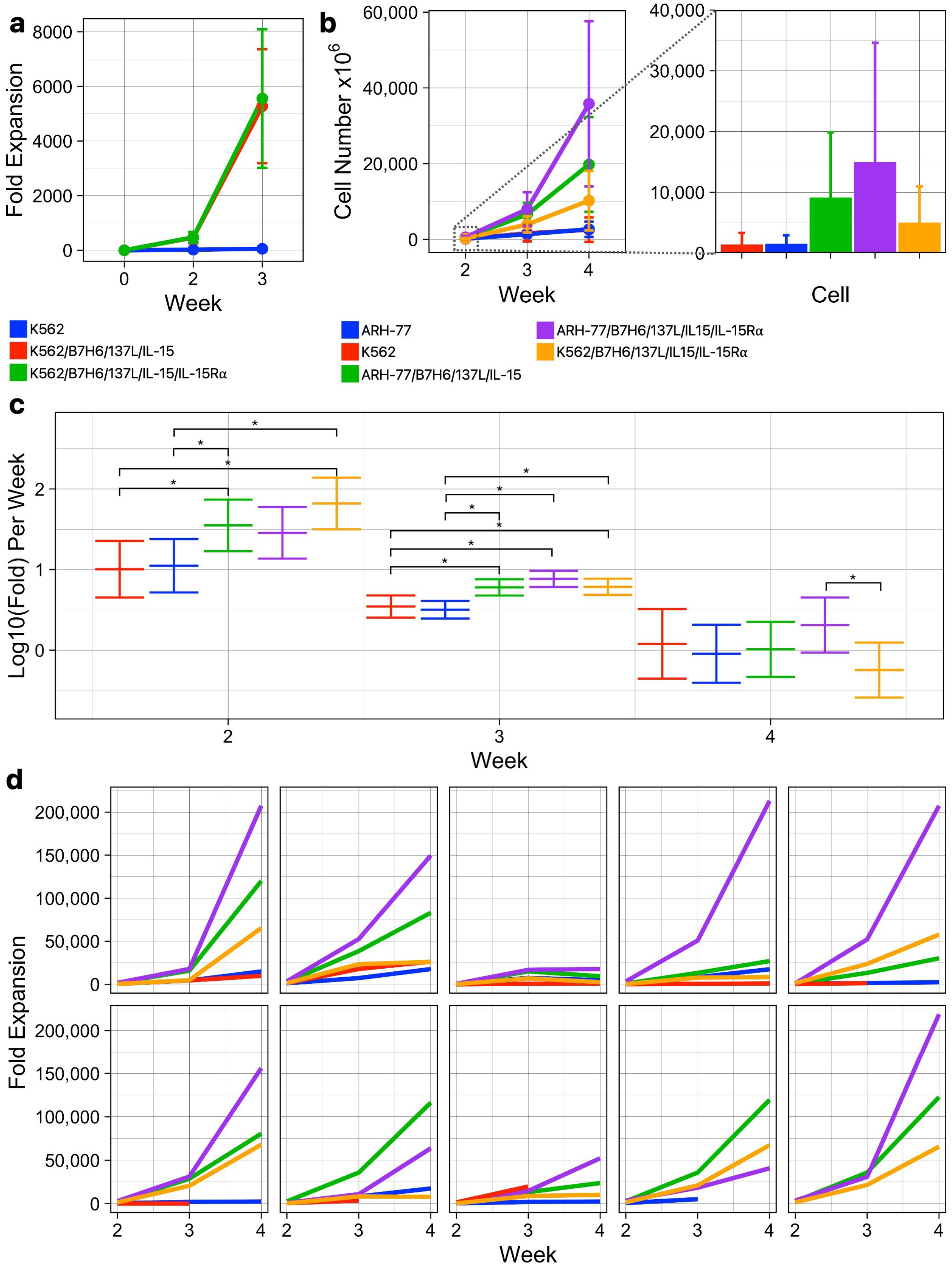

Figure S6. NK Cell Expansion with Modified Feeder Cells. Corresponding to Figure 5. In DME M/F12, PBMCs (n=10 donors) from healthy donors were cultured with various genetically engineered feeder cells to assess their impact on NK cell expansion. (A) Pilot comparison of NK cell fold expansion following co-culture with engineered K562-based feeder cell variants. (B) Absolute NK cell yields over time, shown as raw cell numbers ($\times 10^6$), for cultures with modified feeder cells. (C) Estimated weekly expansion rates derived from a linear mixed-effects model of log10-transformed fold expansion. The model included a second-order polynomial term for week and random slopes for donor and

donor–feeder combinations. Expansion rates, defined as the local slope of log10(fold expansion) with respect to week, are shown for weeks 2, 3, and 4. Adjusted $P < 0.05$ is indicated by *. (D) NK cell fold expansion trajectories over time for individual donors, with each facet representing a single donor.

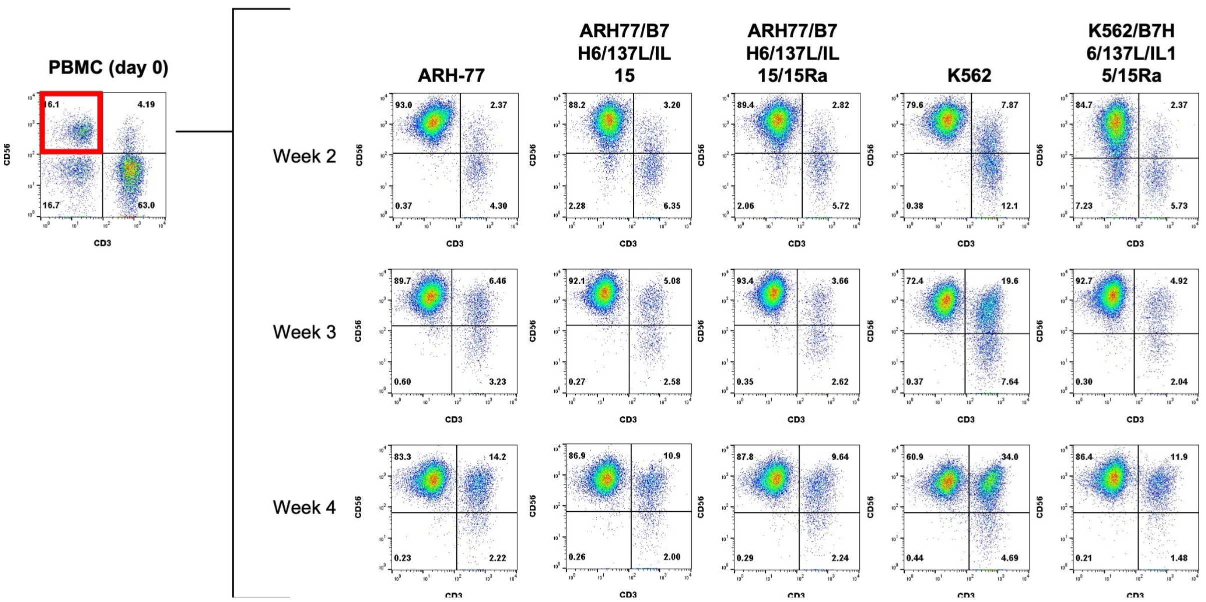

Figure S7. Representative Purity of NK Cells Expanded with Modified Feeder Cells. Corresponding to Figure 5. PBMCs (n=10 donors) from healthy donors were cultured in DMEM/F12 with various genetically engineered feeder cells to assess their impact on NK cell expansion. Representative flow cytometry plots depict the distribution of $CD3^{-}CD56^{+}$ NK cells and other CD3/CD56 expressing populations at the indicated time points.

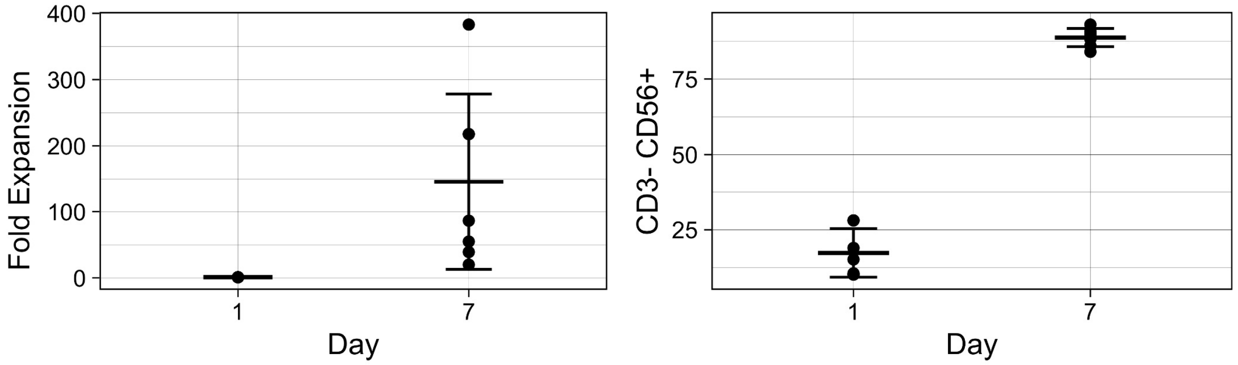

Figure S8. Seven- Day Expansion with ARH77-CD137L-B7H6-IL15-IL15Rα. PBMCs were cultured in DMEM/F12 supplemented with cytokines and stimulated with irradiated ARH77/B7H6/137L/IL-15/IL-15Rα feeder cells. After 7 days, the (A) fold expansion and (B) purity of $CD3^{-}CD56^{+}$ NK cells were quantified by flow cytometry. Each data point represents an individual donor; bars denote mean ± SD.

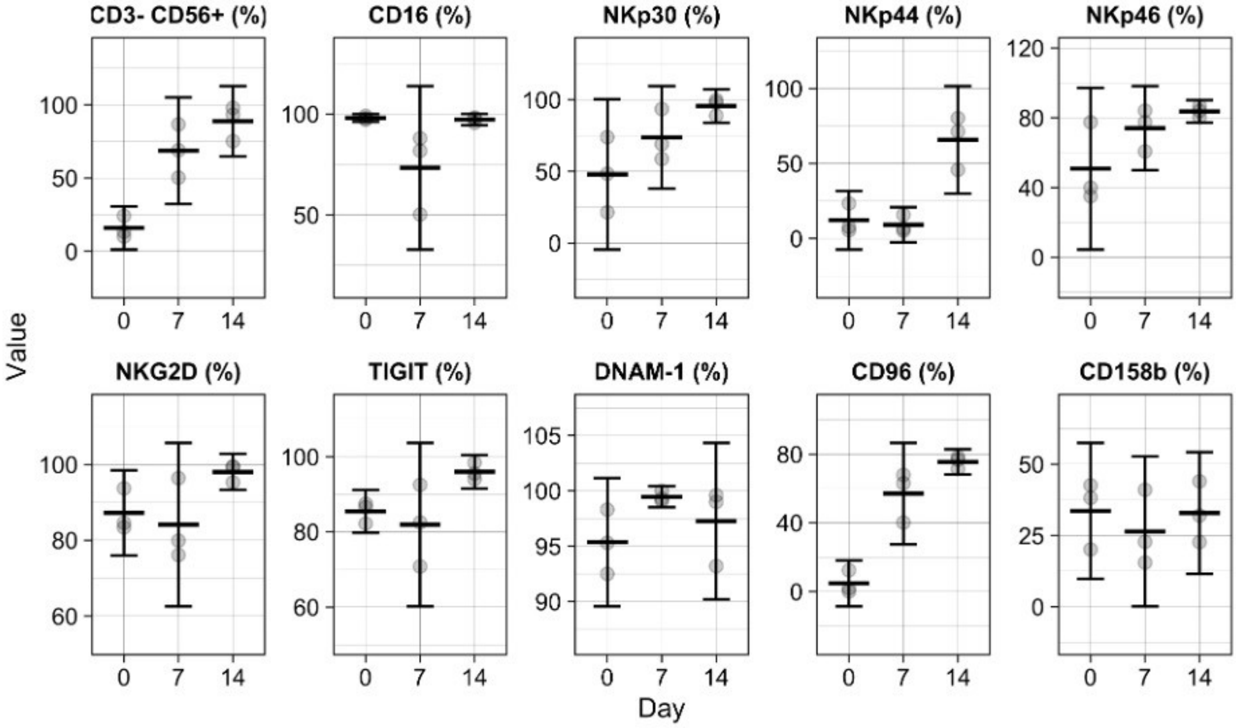

Figure S9. Phenotype of NK Cells Expanded with ARH77-CD137L-B7H6-IL15-IL15Rα. NK cells were expanded from donor PBMCs (n=3) for 2 weeks using ARH77-CD137L-B7H6-IL15-IL15Rα feeder cells in cytokine-supplemented media. Surface expression of indicated markers was assessed by flow cytometry and is presented as the proportion of $CD3^{-}CD56^{+}$ NK cells positive for each marker. Each data point represents an individual donor; bars indicate mean ± SD.

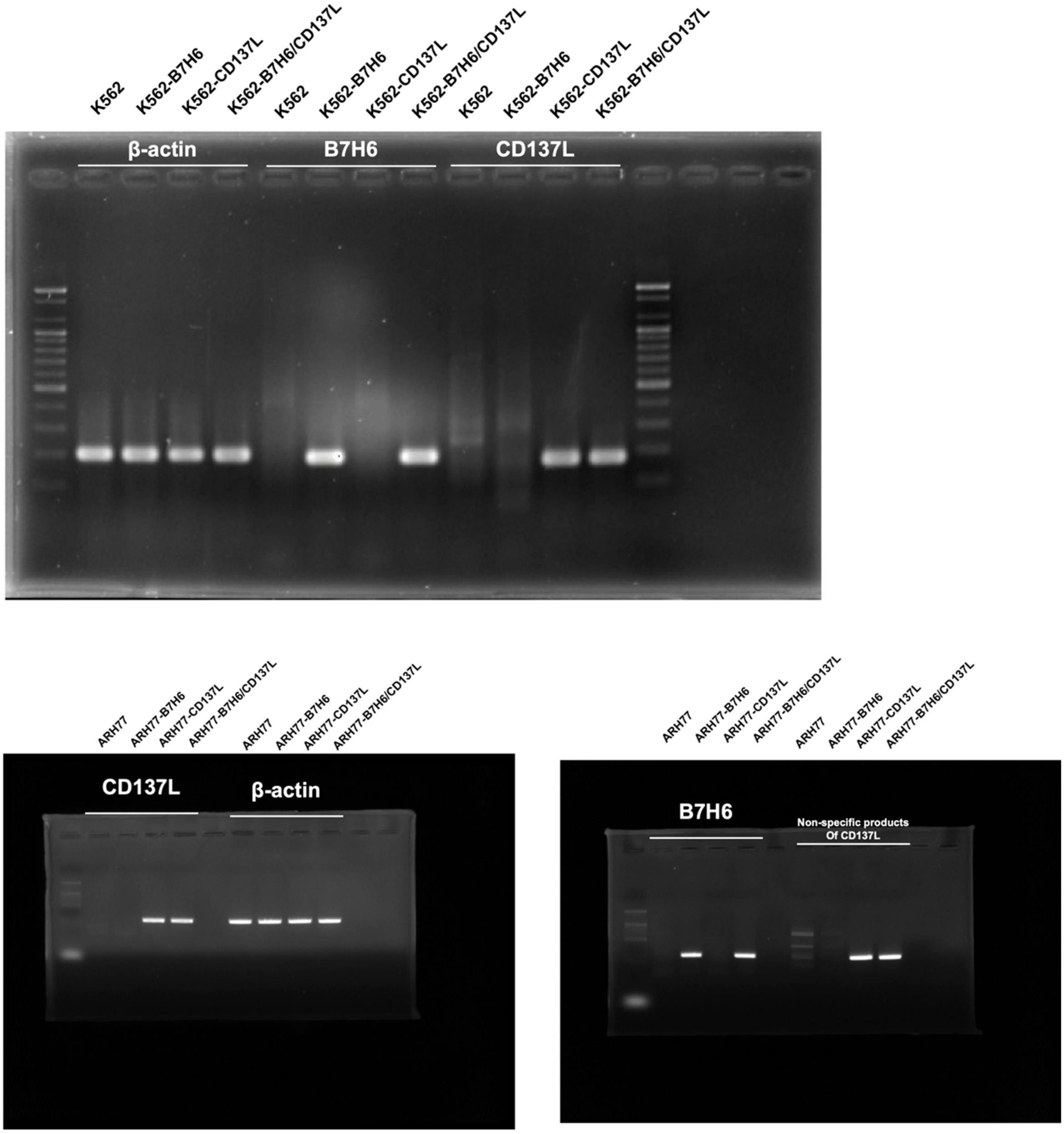

Figure S10. Uncropped RT-PCR gel images corresponding to Figure 3c,d. Full-length uncropped gel images show RT-PCR analysis of CD137L and B7-H6 expression in parental and genetically engineered K562 and ARH-77 feeder cells. β-actin was used as an internal control. The cropped gel images presented in Figure 3c,d were derived from the original uncropped gels.
